# Perceptions of enhanced weathering as a biological negative emissions option

**DOI:** 10.1098/rsbl.2017.0024

**Published:** 2017-04-05

**Authors:** Nick F. Pidgeon, Elspeth Spence

**Affiliations:** 1 Understanding Risk Research Group, School of Psychology, Cardiff University and the Leverhulme Centre for Climate Change Mitigation, Cardiff CF10 3AT, UK; 2 Leverhulme Centre for Climate Change Mitigation, University of Sheffield, Sheffield S10 2TN, UK

**Keywords:** public perceptions, enhanced weathering, biological negative emissions

## Abstract

This paper addresses the social acceptability of enhanced weathering, a technology that would involve spreading silicate particles over terrestrial surfaces in order to boost the biological processes that currently sequester CO_2_ as part of the earth's natural carbon cycle. We present the first exploration of British attitudes towards enhanced weathering, using an online survey (*n* = 935) of a representative quota sample of the public. Baseline awareness of weathering was extremely low. Many respondents remained undecided or neutral about risks, although more people support than oppose weathering. Factors predicting support for weathering and its research included feelings about the technology and trust in scientists. Over half of the sample agrees that scientists should be able to conduct research into effectiveness and risks, but with conditions also placed upon how research is conducted, including the need for scientific independence, small-scale trials, strict monitoring, risk minimization and transparency of results. Public engagement is needed to explore in more detail why particular individuals feel either positive or negative about weathering, and why they believe particular conditions should be applied to research, as part of wider responsible research and innovation processes for biological and other types of negative emissions technologies.

## 1. Introduction

With rising concern about the efficacy of efforts to limit climate change to 2°C, scientists have begun to consider proposals for carbon dioxide removal (CDR) technologies. These range from using energy crops with carbon capture and storage technology, through to direct utilization of captured carbon dioxide in useful chemicals and materials. CDR technologies were reviewed by the Royal Society in their report on climate geoengineering [1], and are based on the idea that we can remove and permanently sequester atmospheric CO_2_ at a planetary scale. The Royal Society report makes a distinction between CDR, which in effect aims to directly reduce one of the key drivers of anthropogenic warming, and the approach of solar radiation management (SRM), which seeks to ameliorate the impacts of warming through artificially altering the earth's radiation balance. Currently the effectiveness, cost and risks of both CDR and SRM approaches to climate geoengineering are highly uncertain [2].

This study focuses upon the CDR technique of enhanced weathering, which would involve boosting the natural biological processes that currently convert and sequester a proportion of atmospheric CO_2_. In a natural chemical reaction occurring between terrestrial plants and organisms on the one hand, and the elements contained in soil on the other, silicate rock weathering releases base cations and forms bicarbonate that eventually find their way into the oceans, where they sequester carbon that can ultimately become locked-in as marine carbonates for long time periods. The idea underlying enhanced weathering is to spread suitable material over agricultural soils, ‘enhancing’ the existing biological weathering process that occurs, potentially also benefiting both crop growth and ocean acidification [3]. CDR technologies have gained in prominence following the Paris International Climate Agreement in 2015, which aspires to a global ‘balance between anthropogenic emissions by sources and removals by sinks of greenhouse gases’ (i.e. net-zero emissions) sometime between 2050 and 2100 [4]. The implication of this is that by then all remaining ‘positive’ fossil fuel emissions (e.g. from aviation, shipping and other hard to decarbonize sectors) must be fully offset by operation of an equivalent set of ‘negative’ emission processes.

Scholars who study the sociology of technologies often characterize proposals for future change such as climate engineering as ‘socio-technical imaginaries’. This is because they represent imagined futures (that may or may not eventually materialize) where the proposed technological changes typically also bring disruptive social, political or environmental consequences. Under such circumstances a prospective technology has to be assessed by society for its wider unintended consequences and ethical implications, alongside conventional assessments of technological risks and economic feasibility. One important consideration here is whether proposals might attract opposition or support among members of the general public, and whether people would wish to place specific conditions upon the development and use of a technology [5].

Studies conducted in the UK demonstrate that, when presented with the opportunity to debate information about climate engineering, members of various publics engage thoughtfully, and at length, with many of the complex issues involved [6–8]. Participants scrutinize these techniques for their hidden assumptions, raising both moral and ethical concerns. For example, people tend to be more concerned about a technology if it is seen as interfering with ‘natural’ biological systems [6]. In addition, climate engineering (and SRM in particular) is often interpreted as a stopgap measure that avoids tackling the root causes of emissions [7,8]. One comparative study conducted with a large online panel sample in Australia and New Zealand [9] explores public perceptions of six climate engineering technologies including enhanced weathering. This finds that weathering had more positive than negative associations, being viewed as having some risk, but was also seen as controllable, not a ‘quick fix’ or artificial, and relatively sustainable over the long-term. However, as compared to CDR by making biochar charcoal from vegetation to lock in CO_2_ (which was viewed very positively by their respondents), and to SRM by placing large mirrors in orbit around the earth to block or reflect sunlight (which was viewed very negatively), enhanced weathering was seen as a relatively indistinct climate engineering approach without strong associations either way. To date, however, there has been no other detailed research into public attitudes towards enhanced weathering in the UK or elsewhere.

## 2. Methods

The study formed part of a larger research survey conducted in April 2016 that was designed to explore perceptions of ocean acidification and climate change risks. Questions about enhanced weathering were asked at the end of the protocol. A nationally representative sample of the British public (*n* = 935) aged 18+ were recruited through a specialist panel company to complete an online survey. There was a spread of ages (18–75), gender (46.4% Male) and levels of science education.

Because very few would have heard about the issue before, a short definition of enhanced weathering was presented to participants onscreen and for a minimum of 30 s, prior to asking the relevant questions (table 1). It included information about the weathering process generally, then explained how ‘enhanced weathering’ would speed this up before outlining the possible impacts on the environment (including possible effects on plants and animals) as well as issues surrounding the transportation and use of the minerals. After reading this information, participants completed seven items assessing their views, some based on previous surveys of public perceptions of climate engineering [11]. These items assessed participants' awareness of weathering, the extent to which they would support it to tackle climate change, their feelings about it, as well as acceptability of risks, and opinion on the balance of risks and benefits. Two final questions opened with a sentence explaining that scientists wanted to conduct research into the technique before asking if the participant was supportive of such research. If they responded ‘not sure’ or agreed that research should proceed they were then asked in an open-ended question regarding whether there were any controls they would wish to see in place before research went ahead.

**Table 1.** Information provided and questions asked.

*Definition of enhanced weathering read by survey participants*
‘Weathering’ is the breakdown of rocks and minerals at the Earth's surface, by the action of rainwater, extremes of temperature, and the contribution of living organisms' activities. Chemical weathering, or chemical breakdown of rocks by rainwater, is an important part of the carbon cycle as carbon dioxide is naturally removed from the atmosphere over thousands of years through this process. Silicate minerals form one of the most common rocks on Earth and they react with carbon dioxide to form carbonate and bicarbonate ions, locking away the carbon dioxide through this chemical reaction. Eventually this will end up being transported to the rivers and into the oceans, where plankton may use these ions to form calcium carbonate (for their shells and skeletons) or these products will get locked away and stored in sediments for a long time.
It has been proposed that speeding up this type of weathering (a technique described as ‘enhanced weathering’) may help to reduce carbon dioxide levels and help combat climate change. Enhanced weathering artificially accelerates the processes described above as rocks are crushed and spread over very large surfaces of the land. Some scientists have proposed that this technique could help reduce the carbon dioxide in the air that is causing climate change. To have any real impact on the world's climate it would have to be done on a very large scale (potentially an effort equivalent to the size of the current oil and gas industry) and over a very long period of time. There will also be impacts of large-scale mining, processing and transport of the minerals to be used, and its precise impacts upon other uses of the land and on plants and living creatures are as yet uncertain.
*Survey items on enhanced weathering*
AWARENESS^a^
Q1. Before today, how much if anything, would you say that you know about enhanced weathering?
(I know a great deal about enhanced weathering, I know a fair amount about enhanced weathering, I know just a little about enhanced weathering, I have heard of enhanced weathering but know almost nothing about it, I have not heard about enhanced weathering before today: coded from 5 to 1.)
SUPPORT
Q2. Overall, to what extent would you support enhanced weathering to tackle climate change? (Strongly support, tend to support, neither support nor oppose, tend to oppose, strongly oppose, don't know: coded 5 to 1.)
AFFECT^a^
Q3. In general, how do you feel about enhanced weathering? (Very negatively, negatively, neither negatively or positively, positively, very positively coded 1 to 5.)^a^
ACCEPTABILITY^a^
Q4. On the whole, how acceptable or unacceptable are the risks of enhanced weathering to you? (Very acceptable, acceptable, neither acceptable nor unacceptable, unacceptable, very unacceptable: coded 5 to 1.)
RISK/BENEFITS^a^
Q5. From what you know or have heard about enhanced weathering, on balance, which of these statements, if any, most closely reflects your own opinion? (The benefits far outweigh the risks, the benefits slightly outweigh the risks, the benefits and risks are about the same, the risks outweigh the benefits, the risks highly outweigh the benefits: coded 5 to 1.)
RESEARCH
Q6. As this technique currently is uncertain, scientists want to conduct research into the effectiveness and risks of this method of removing carbon dioxide from the air. Do you think this research should be carried out? (Not at all, probably not, no opinion, probably yes, definitely: coded 1 to 5.)
Q7. If you think research should be done, are there any controls you might wish to see placed on the research or the scientists before it went ahead? (Open-ended.)
*Additional survey items asked of participants and used in data analysis*
GENDER
Male, female, prefer not to say. (Coded 1, 2, 3, missing.)
EDUCATION
What is the highest level of science-based education that you have? (No formal science qualifications, GCSE/O Level/Standard Grades, A-Level/Higher/BTEC, vocational/NVQ, degree or equivalent, postgraduate qualification, other: coded 1 to 6, missing.)
CONCERN ABOUT CLIMATE CHANGE
How concerned, if at all, are you about climate change (sometimes referred to as global warming)? (Very concerned, fairly concerned, not very concerned, not at all concerned, don't know, no opinion: coded 6 to 3, missing.)
TRUST SCIENTISTS^a^
To what extent do you agree or disagree with the following statement? We can trust scientists to tell the truth about climate change. (Strongly agree, tend to agree, neither agree nor disagree, tend to disagree, strongly disagree: coded 5 to 1.)
CLIMATE CHANGE A GOVERNMENT PRIORITY^a^
How high or low a priority should it be for the UK government to take action on climate change? (Very low priority, fairly low priority, medium priority, fairly high priority, very high priority: coded 1 to 5.)

^a^Methodological note: Several of the questions, as indicated above, omit the ‘don't know’ option, since this will encourage a respondent to think a little more deeply about the question asked. Although one might expect, where prior awareness of an issue is very low as here, that ‘don't know’ categories are necessary in order to avoid expressions of ‘pseudo-opinion’, evidence shows that such responses differ little between scales that have, and those that omit, this option [10].

## 3. Results

Very few people reported that they were aware of the idea of weathering. 70.3% stated they had not heard about this before undertaking the survey, with only 6.5% stating they knew either a great deal or a fair amount. There was more support (37.2%) than opposition (16.8%) for the technique, although the most common response to this question was to neither support nor oppose (46.0%). The latter response can reflect a number of sentiments, including ambivalence (people could perceive counterbalancing pros and cons) as well as neutral, uninformed or indifferent stances.

The next three questions gauged views on acceptability. Again, the most frequent answer selected was the middle response (57.0% felt neither negative nor positive; 55.2% judged the risks neither acceptable nor unacceptable; and 41.6% felt the balance of benefits against risks were about the same). For those who did express an opinion, slightly more people were positive (22.8%) than were negative (20.1%), felt the risks were acceptable (25.6%) than felt they were unacceptable (19.2%), and that the benefits outweighed the risks (33.9%) compared with those who felt risks outweighed benefits (24.5%).

The final questions asked about conducting research. 53.3% of the sample said it should probably or definitely be allowed. Only 9% thought it should not be allowed. The follow-up question probed whether respondents wanted particular controls to be placed on the research or the scientists involved. Of those asked this question (a total of 840 respondents), many reported being unsure about controls (18.9%) or that no controls were required (17.0%). The remaining answers were grouped into six broad categories: that the research should proceed such that *risks to the environment, animals and humans should be absent or minimized* (10.2% who answered mentioned this); that the research should be done on a *small-scale, or in a location* where its impacts would be minimized (8.7%); that the research needed to be *bias-free and independent* of profit-making and corporate (or sometimes government) interests (5.6%); that there should be *rigorous monitoring* of trials (4.9%); that the findings of the research should be *transparent and open* to anybody to see (2.9%); and finally, that *experts should be left to decide* on controls (1.4%). In some instances a respondent gave a detailed rationale that could be coded under several of the above categories. For example, one participant wrote that ‘provided it is done on a very small scale, careful on the amount of energy used, with close monitoring down a stretch of river to the estuary and out to sea avoiding any SSSIs (sites of special scientific interest) etc. then this should go ahead over a long time scale. Particular note should be made of any effects on marine and freshwater life by increased particulates and plankton blooms which could blanket some areas’.

We use regression analysis (table 2) to explore if theoretically relevant variables (gender, concern about climate change, support for government action on climate change, trust in scientists, perceived risks and benefits of weathering) would predict an individual's support for weathering (Q2) and whether research should go ahead (Q6).

**Table 2.** Regression analyses. *r* corresponds to Pearson's correlation coefficients and *B* to standardized beta coefficients. *R*^2^ is the proportion of variance in support for weathering and research respectively explained by all predictor variables in each model. The *p* value shown is the probability of obtaining the observed result when no true effect exists. *F* is the test statistic for the analysis of variance ratio.

	support for weathering^a^		support for research^b^	
predictor variables	*r*	B	*r*	*B*
gender	−0.03	−0.02	−0.03	0.08
education	−0.01	−0.03	0.06	0.08
prior knowledge of weathering	0.21	0.14**	−0.04	−0.10*
feeling about weathering	0.69	0.36**	0.39	0.25**
acceptability of risks	0.67	0.20**	0.34	0.04
benefits outweigh risks	0.67	0.28**	0.35	0.12*
concern about climate change	0.23	0.03	0.25	0.05
climate change government priority	0.19	0.04	0.29	0.14*
trust scientists on climate change	0.30	0.06*	0.28	0.12**

For regressions, ^a^*R*^2^ = 0.64** (*F* = 167.15), ^b^*R*^2^ = 0.25** (*F* = 32.20).

*p < 0.01, **p < 0.001.

Whether people felt positive about the idea of weathering (termed ‘positive affect’), risk acceptability, perceived benefits exceeding risks, as well as trust in climate scientists to tell the truth about climate change all significantly predicted support, and collectively accounted for almost two thirds of variance in the support item (*r*^2^ = 0.64, *F* = 167.15, *p* < 0.001). Although we had expected that those concerned about climate change would tend to favour solutions proposed by scientists, this did not independently predict support in the regression.

A slightly different pattern emerges when we look at whether research should proceed. Here positive affect, benefits outweighing risks and trust in scientists all significantly predict support for research. Additionally, the extent to which people feel that the government should prioritize acting on climate change is a significant predictor of support for research, with personal concern about climate change again not significant. Finally, prior knowledge of weathering was a weak (and contradictory) predictor in the two regression analyses.

## 4. Discussion

Unsurprisingly, very few in this study had heard of enhanced weathering, although this may change in the future if negative emissions become more prominent in media and policy discussions of climate change. Accordingly, our findings serve as important baseline measures. The modal response on the risk perception items was neutral or ambivalent, although of those who expressed a preference somewhat more people thought benefits would outweigh risks. The finding here that a positive feeling is the most potent predictor of support for the technology is in line with existing research on the powerful role that affect, or feeling, plays in perceived risks [12]. Likewise, people's trust in scientists is known to underpin many risk acceptability judgements [13]. These findings are in line with research showing that CDR approaches to geoengineering tend to be viewed in a more favourable light than SRM [11], and are also fully consistent with the study conducted in Australia and New Zealand by Wright *et al*. [9]. The latter likewise report more positive than negative associations with enhanced weathering, albeit these beliefs, as also found here, were relatively neutral or are currently indistinct. All of this suggests that scientists and regulators should take particular care to ensure that enhanced weathering, if ever developed as a biological negative emissions technique, can deliver its promised benefits while also guarding against the emergence of unanticipated risks to ecosystems or human populations. Meeting both of these conditions should also serve to maintain the trust of the public.

Support for research was found to be much stronger than support for the technique itself, implying that people distinguish between development and deployment. While research might be therefore allowable, this does not necessarily mean people endorse full-scale deployment (also [8]). Alongside the standard risk perception variables and trust, support for research was also dependent upon people believing that the government should take action on climate change, suggesting that proposals for weathering research should not be separated from the wider debates about climate change mitigation. A number of our respondents wanted research that does take place to be independent of corporate interests, with initial small-scale trials, strict monitoring, minimization of risks to ecosystems and transparent reporting. These conditions are consistent with the Oxford Principles for governance of climate engineering research [14], and proposals for responsible research and innovation processes with emerging technologies more generally [15].

Surveys such as this are, however, always a blunt instrument for exploring public views of complex science issues. Given the low prior awareness among respondents, the responses obtained do have to be interpreted carefully in the light of the description provided—which was relatively technical in nature so as to reflect a broad scientific understanding of the weathering process and its impacts. We know that different information ‘frames’ can influence responses to unfamiliar technology descriptions [16,17], hence further research is needed to understand if and how different information (e.g. natural versus unnatural, risk verses benefit frames) will influence responses to enhanced weathering and other negative emission proposals. In-depth public engagement, which typically allows participants to explore and debate quite disparate technical information and ethical arguments as one means of developing ‘informed’ preferences and opinions [16], would also be desirable. In particular, engagement could be conducted in locations where enhanced weathering technologies might be deployed at scale, such as major crop growing regions, as well among populations who are the most responsible for, and conversely most impacted by, anthropogenic emissions. In this way people's fears, hopes and ethical concerns about the scientific and social visions that this new emerging technology might bring can be more fully explored.

## Data accessibility

The data are deposited in Dryad: (http://dx.doi.org/10.5061/dryad.53v1r) [18].
